# Testing P2Y12 platelet inhibitors generics beyond bioequivalence: a parallel single-blinded randomized trial

**DOI:** 10.1186/s12959-022-00405-y

**Published:** 2022-08-17

**Authors:** Bassem Zarif, Lamyaa Soliman, Nirmeen A. Sabry, Eman Said

**Affiliations:** 1 Department of Cardiology, National Heart Institute, Cairo, Egypt; 2 Department of Clinical and Chemical Pathology, Faculty of Medicine, Cairo University, Cairo, Egypt; 3 Department of Clinical Pharmacy, Faculty of Pharmacy, Cairo University, Kasr Alainy Street, Cairo, 11562 Egypt

**Keywords:** Ticagrelor. Generics, Acute coronary syndrome

## Abstract

Cardiovascular diseases are the leading cause of death worldwide. Ticagrelor is an oral antiplatelet drug used in acute coronary syndrome. Although generic drugs are approved for their bioequivalence to the original product, they are not necessarily to be therapeutically equivalent. This study was conducted to prove the efficacy and safety of ticagrelor generically named Ticaloguard® compared to its brand Brilique® in healthy volunteers. A loading dose of 180 mg ticagrelor named Brilique® or Ticaloguard® followed by a 90 mg twice daily regimen as maintenance dose was given to 14 and 15 volunteers in Tica and Brili groups, respectively. The platelet aggregation on the ADP agonist was assessed at baseline and repeated 1 h and 3 h after the loading dose, on day 4 (after reaching steady-state), 12 and 24 h after discontinuation of the antiplatelet drug. Adverse effects from trial medications were noted by direct questions. It was shown that generic Ticaloguard® provides a similar therapeutic effect and safety as its branded Brilique® (*p* > 0.05). This will permit safe and trusted use of the generic Ticaloguard® when treating it in the same manner as Brilique®. Testing generic drug effects rather than simple bioequivalency, especially for drugs that are used in critical life-threatening situations, is crucial. We advocate applying this form of a clinical trial to test surrogate clinical efficacy for generics used in critical indications before having real-world data whenever possible.

## Purpose

Although generic drugs are approved for their bioequivalence to the original product, they are not necessarily therapeutically equivalent. The aim of our study is to introduce the concept of “studying the clinical effectiveness” of generic drugs in comparison to the brand drug in a randomized clinical trial (RCT) rather than simply comparing “their bioequivalence” to build more trust in to use of the generic drug. This study was conducted to prove the efficacy and safety of ticagrelor—generically named Ticaloguard® compared to its brand Brilique® in healthy volunteers. We studied the effect of both drugs on ADP-induced platelet aggregation by a light transmission aggregometer. Adverse effects from trial medications were noted by direct questions. We advocate applying this form of a clinical trial to test surrogate efficacy for generics used in critical indications.

## Introduction

Cardiovascular diseases (CVDs) are still the leading cause of death in Egypt and worldwide [1]. Myocardial infarction and stroke counted for more than 80% of global CVD deaths, 75% of them occurred in low- and middle-income countries [2]. Acute coronary syndromes (ACS), presented as acute myocardial infarction with or without ST-segment elevation and unstable angina, are clinical status induced by the thrombus formation following the disruption of unstable atherosclerotic plaque [3]. Acute coronary syndromes (ACS) have a yearly incidence of at least 600,000 patients in the United States, the majority of whom undergo coronary angiography with percutaneous coronary intervention (PCI) [4]. ACS are a major health care burden that require early diagnosis, effective therapy to reverse ischemia, restore normal coronary blood flow, and minimize myocardial damage [5].

Dual antiplatelet therapy with aspirin and a platelet P2Y_12_ receptor antagonist (P2Y_12_ inhibitor) is the treatment of choice for the prevention of atherothrombotic events in patients with acute coronary syndromes (ACS) [6].Clopidogrel, prasugrel, and ticagrelor are the most commonly used oral platelet P2Y_12_ inhibitors. [7].

Ticagrelor is a potent direct-acting, reversibly binding P2Y12 platelet receptor antagonist which is used to prevent atherothrombosis events in patients with ACS especially those who have received coronary stents [8]. Ticagrelor has many advantages over the commonly used antiplatelet, clopidogrel; First, it has a rapid onset of action as it does not require metabolic activation with peak activity in approximately 2 h (hrs) after the loading dose [9]. Second, it is a more effective inhibitor of adenosine diphosphate (ADP)-induced platelet aggregation than clopidogrel [10]. Third, the platelet inhibition of ticagrelor is temporary due to its short half-life (8 to 12 h) combined with the reversible receptor binding [11, 12]. Current guidelines support the preferential use of prasugrel and ticagrelor over clopidogrel -in many clinical situations- because of their superior net clinical benefits. [13].Ticagrelor is available in the market as oral tablets with the brand-name drug Brilinta/Brilique® [14]. After the patent of the brand-name equivalent expires, less expensive generics are produced by many manufacturers [15]. Generic drugs contain the same active ingredients as the original brand with identical or acceptable bioequivalent pharmacokinetic and pharmacodynamic characteristics [16]. Generics are of similar efficacy, and safety as brand-name medicine and they should provide the same clinical benefit [16]. Due to the major and disruptive shortfalls in financing in low- and middle-income countries especially after the coronavirus disease of 2019 (COVID-19) pandemics [17]; it seems necessary to prove the efficacy of generic medicines to promote their use and thus lowering total pharmaceutical expenditures. Therefore, the aim of this study was to introduce the concept of “studying the clinical effectiveness” of generic drugs in comparison to the brand drug rather than simply comparing “their bioequivalence”, To build more trust in the use of generic drugs; this study was conducted to prove the efficacy and safety of ticagrelor generically named Ticaloguard® compared to its brand Brilique® in healthy volunteers by comparing the effectiveness of platelet function inhibition of both drugs using the light transmission assay.

## Methods

### Study design and sample size calculation

This was an interventional parallel, single-blinded randomized trial. This study was designed to be a pilot trial. The trial followed the rule of 12 which recommend at least 12 participants per group for pilot studies [18, 19]. 10% expected drop-out was also calculated.

#### Inclusion criteria

Healthy adult volunteers of both sexes, aged between 18–64 years old with no previous blood disease or bleeding diathesis were recruited. Volunteers should fill out a written consent before the study.

#### Non-inclusion criteria

Volunteers with any abnormalities in the complete blood count (CBC) at entry time, those who had any medical conditions that made antiplatelet therapy contraindicated, and those who had used aspirin, non-steroidal anti-inflammatory drugs (NSAIDs), or any over the counter medications (OTC) that contain medication such as antihistamines, antibiotics within the previous month [20]. In addition, those with active bleeding, a history of intracranial hemorrhage, a hypersensitivity history to the medication, moderate to severe hepatic impairment, and the concomitant use of strong CYP3A4 inhibitors [21].

### Study randomization

To determine the study allocation for the recruited volunteers, simple randomization has been carried out [22]. Shuffled cards with random numbers, from 1:30, were randomly distributed to volunteers. Volunteers were then allocated into two groups based on their odd or even selection.

### Ethics approval

All volunteers were enrolled from Cairo, Egypt in November 2021. The study protocol was approved by the Research Ethics Committee, Faculty of Pharmacy, Cairo University, Egypt with a serial number (CL30101). Written informed consent was signed by all volunteers prior to the study. This trial was registered at clinicaltrial.gov.

### Clinical intervention

Eligible volunteers were randomly allocated (1:1) to either one of two groups: volunteers in the Brilique (Brili) group received a loading dose of 180 mg brand Brilique® (AstraZeneca AB, Gartunavagen, SE- 15,185 Sodertalje, Sweden) then 90 mg twice daily regimen for 4 days, while volunteers in the Ticaloguard (Tica) group received the Egyptian made generic ticalogaurd® (Marcyrl Pharmaceutical Industries, Cairo, Egypt). All volunteers were instructed to avoid food containing onion or garlic, caffeinated drinks like tea, coffee, soda, chocolate, etc., and cigarette smoking four days before and throughout the study period [23–25].

All subjects were screened for their demographic and baseline clinical characteristics before conducting the study.

### Medication efficacy

Platelet aggregation on ADP agonist and whole blood count (CBC) were assessed at baseline and repeated 1 h and 3 h after the loading dose (to test onset and peak of drug action), at day 4 (after reaching steady-state), 12 and 24 h after discontinuation of the antiplatelet drug (to evaluate the declining action of the medication).

### Compliance to medications

Compliance was assessed every visit by counting the remaining number of tablets. To increase the participants’ compliance, volunteers were followed up via a daily telephone call. Volunteers who completed the study had drug compliance of 100%.

### Medication safety

Adverse effects s from trial medications including the incidence of major/minor bleeding events, new-onset dyspnea, arrhythmia and/or any other events reported by the subjects were noted by direct questions. Major bleeding events were defined as fatal bleeding or overt bleeding with a drop in hemoglobin level of at least 20 g/L or requiring transfusion of at least 2 units of packed blood cells [26]. Minor bleeding events were defined as acute clinically overt events not meeting the criteria for either major or clinically relevant non-major bleeding [27].

### Blood sampling and parameters assay

After a fasting period of approximately 8 h, a 5 mls venous blood sample was drawn aseptically from the healthy volunteers and divided into sodium citrate (blue capped) tube for platelet function testing and an Ethylenediaminetetraacetic acid (EDTA) purple top tube for complete blood count (CBC). Blood samples were processed within an hour of blood collection. The CBC analysis was performed with the AU680 Beckman Coulter auto- chemistry analyzer, USA. In the platelet function test, the whole-blood specimens were centrifuged for 10 (minutes) min at 600 rpm to obtain platelet-rich plasma (PRP). Platelet aggregation was performed on the CHRONO-LOG platelet aggregometer (Chrono-Log Corporation, Havertown, USA) using an adenosine diphosphate (ADP) agonist at 10 μmol. The tube containing platelet-rich plasma (PRP) was inserted in between a light source and a photocell, and then the ADP agonist was added. As the platelet aggregate, the light transmission increased proportionally. Light Transmission Aggregometry (LTA) was repeated 1 h and 3 h after the loading dose, on day 4, and finally 12, and 24 h after discontinuation of the antiplatelet drug. All the analyses were performed at the clinical pathology department, Kasr Alainy hospital, Cairo, Egypt.

### Statistical analysis

IBM statistical package for social science (SPSS) version 25 was used for statistical analysis. Continuous data were expressed as median (range) or mean ± standard deviation (SD) while categorical variables were presented as frequencies and/or percentages. For non-parametric comparison, the Mann–Whitney test was used for detecting differences between 2 independent groups while Fisher’s exact was applied for nominal data. The two-sided *p*-value ˂ 0.05 was considered significant for all statistical purposes [28]**.**

## Results

**Fig. 1:**
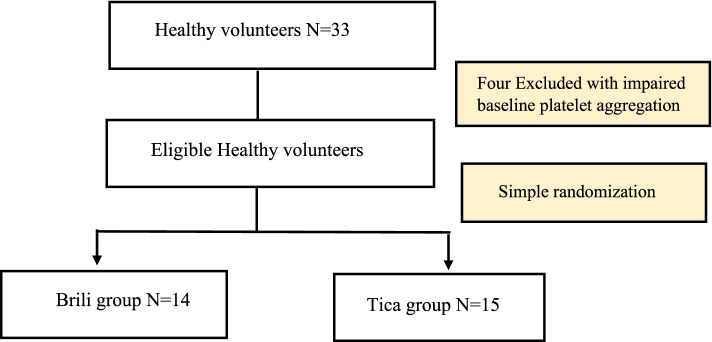
Volunteer flow chart. Abbreviation: Brili, Brilique®; Tica, Ticaloguard®

After screening 33 apparently healthy volunteers for inclusion and exclusion criteria, twenty-nine of them (8 male, 21 female) were eligible and completed the study; four of which were excluded as they had defective baseline platelet aggregation. A volunteer flow chart is presented in Fig. 1. Baseline demographics and clinical characteristics of study volunteers were comparable between the two groups Table 1.

**Table 1:** Comparison between volunteers in Brili and Tica groups on different demographic and baseline laboratory parameters

Parameter	Brili group-brand (*N* = 14)	Tica group-generic (*N* = 15)	*p*-value
No of males (%)	3 (21.4%)	5 (33.3%)	0.682^¥^
Age (years)	38.36 ± 7.53	38.80 ± 8.21	0.881^#^
BMI (Kg/m^2^)	31.44 ± 4.95	30.29 ± 4.40	0.513^#^
ALT (IU/L)	20.21 ± 9.61	19.33 ± 8.61	0.797^#^
Albumin (g/L)	4.20 ± 0.20	4.26 ± 0.28	0.530^#^
Scr (mg/dL)	0.72 ± 0.12	0.78 ± 0.14	0.239^#^
Hb (10^6^cell/μL)	12.34 ± 1.68	12.67 ± 1.45	0.575^#^
PLT (10^3^cell/μL)	297 ± 82	314 ± 111	0.637^#^
ADP Platelet aggregation (%)	69 (48–81)	75 (48–85)	0.06^$^
Previous bleeding during or post-surgery (%)	1 (7.14)	0 (0)	0.483^¥^

Data are presented as median (range) or mean ± SD for continuous variables and frequency (percentage) for categorical variables

*Abbreviation*: *Brili* Brilique®, *Tica* Ticaloguard®, *BMI* Body mass index, *ALT* Alanine transaminase enzyme, *Scr* Serum creatinine, *Hb* Hemoglobin, *PLT* Platelet count, *ADP* Adenosine diphosphate. P#: Non-significant by independent T-test P$: Non-significant by independent-sample Mann–Whitney U test; P¥: Non-significant by Fisher’s Exact test

### Efficacy of trial medications

**Fig. 2:**
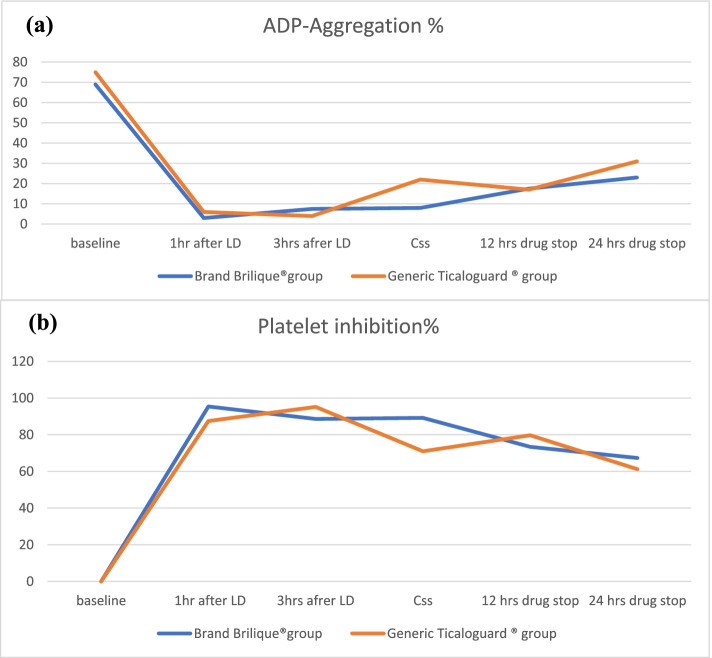
(**a**) Effect of generic Ticaloguard® and brand Brilique® on ADP aggregation and (**b**) platelet inhibition at different time intervals. Abbreviations: LD, loading dose; Css, steady-state concentration

As shown in tables 2 and 3 baseline aggregation test using ADP agonist and the % platelet inhibition were comparable between the two groups and remained non-significant shortly after 1 h and 3 h of the loading dose, at day 4 after reaching steady state, 12 and 24 h of the medication discontinuation in both Brili and Tica groups (*p* > 0.05). A summary of the generic and brand ticagrelor effects at different time intervals is presented in Fig. 2.

**Table 2:** Effects of a 180 mg loading dose followed by 90 mg maintenance dose of Brilique ® versus Ticaloguard ® on ADP aggregation test of healthy volunteers at different time intervals

Groups	% ADP aggregation					
	Baseline	1 h after LD	3 h after LD	At day 4, reaching Css	12 h after drug stop	A day after drug stop
Brili group (*n* = 14)	69 (48–81)	3 (0–13)	7.5 (0–87)	8 (1–33)	17.5 (0–36)	23 (0–48)
Tica group (*n* = 15)	75 (48–85)	6 (0–80)	4 (0–88)	22 (0–29)	17 (5–35)	31 (10–75)
*p*-value	0.06	0.067	0.721	0.189	0.844	0.169

Data are presented as median (range)

*Abbreviation*: *Brili* Brilique®, *Tica* Ticaloguard®, *ADP* Adenosine diphosphate, *LD* Loading dose, *hr* Hour, *Css* Steady state concentration. P: Non-significant by independent-sample Mann–Whitney U test

**Table 3:** Effects of a 180 mg loading dose followed by 90 mg maintenance dose of Brilique® versus Ticaloguard® on % platelet inhibition compared to zero inhibition at baseline of healthy volunteers at different time intervals

Groups	% Platelet inhibition				
	1 h after LD	3 h after LD	At day 4, reaching Css	12 h after drug stop	A day after drug stop
Brili group (*n* = 14)	95.4 (72.9–100)	88.6 (-12.9–100)	89.2 (43.1–98.5)	73.4 (39.5–100)	67.3(34.2–100)
Tica group (*n* = 15)	87.5 (-2.5–100)	95.2 (-12.8–100)	71.0 (56.2–100)	79.7 (50–92.7)	61.25 (-20.6–86.6)
*p*-value	0.85	0.911	0.371	0.76	0.315

Data are presented as median (range)

*Abbreviation*: *Brili* Brilique®, *Tica* Ticaloguard®, *LD* Loading dose, *hr* Hour, *Css* Steady state concentration. P: Non-significant by independent-sample Mann–Whitney U test. Negative values indicate resistant cases

### Safety of trial medications

Neither minor nor major bleeding events were detected in both treated groups. Two volunteers in Brilique® group complained of new onset dyspnea. Three incidences of dyspnea at bedtime described as remaining, tolerable dyspnea, almost resolved after stopping the medication was reported by one volunteer. The other patient’s dyspnea was also at bedtime but once and resolved spontaneously after a few minutes. No dyspnea was detected in volunteers who received Ticaloguard®. On the other hand, a minor rash resolved spontaneously was reported in two volunteers who received Ticaloguard and one in the Brilique group. No other adverse effects s from medications were recorded during the study period. Both hemoglobin (Hb) level and platelets (PLT) count remained comparable after the study period (Table 4).

**Table 4:** Comparison on incidence of side effects developed in Brili versus Tica groups during the study period

Parameter	Brili group-brand (*N* = 14)	Tica group-generic (*N* = 15)	*p*-value
No of males (%)	3 (21.4%)	5 (33.3%)	0.682^¥^
New-onset dyspnea (%)	2 (14.2%)	0	0.224^¥^
Rash (%)	1 (7.1%)	2 (13.3%)	0.999^¥^
Hb (10^6^cell/μL)	12 (9.2–15.4)	12 (9.6–15.1)	0.982^#^
PLT (10^3^cell/μL)	315 (172–405)	303 (181–484)	0.836^#^

Data are presented as median (range) for continuous variables and frequency (percentage) for categorical variables

*Abbreviation*: *Brili* Brilique®, *Tica* Ticaloguard®, *Hb* Hemoglobin, *PLT* Platelet count, P#: Non-significant by independent-sample Mann–Whitney U test; P¥: Non-significant by Fisher’s Exact test

## Discussion

The uncertain global economy and the rising expense of medication burden the healthcare expenditures [29, 30]. Generic drugs provide an efficient allocation of financial resources which, will help save on healthcare costs [31, 32]. Although generic drugs are approved for their bioequivalence to the original product, they are not necessarily to be therapeutically equivalent [29]. Physicians need to be assured when prescribing generic cardiovascular drugs indicated for patients with serious conditions and the health care system will endorse their larger use [33]. Generic drugs should be tested to prove their therapeutic response. In the present study and to the best of our knowledge, for the first time, the efficacy and safety of a generic ticagrelor (Ticaloguard®) to its brand (Brilique®) were compared. It was shown that generic Ticaloguard® provides a similar therapeutic effect to its branded Brilique®.

The generic Ticaloguard ® achieved marked platelet inhibition within 60 min after a loading dose (LD). This early and strong onset of PLT inhibition is highly desirable in patients undergoing primary percutaneous coronary intervention (PCI), especially for interventions in an acute situation [34].

Following a 90 mg twice daily regimen as a maintenance dose, Ticaloguard maintained a similar therapeutic effect as the branded Brilique®. This was confirmed by a comparable platelet inhibition early at onset and when reaching steady-state concentration (C_SS_). This finding is extremely important to avoid the phenomenon of subacute stent thrombosis [35].

Increased bleeding risk, which may last for days after stopping the oral P2Y12 receptor antagonists [36], possesses a great challenge for the establishment of hemostasis in patients with major bleeding or those who require emergency invasive procedures whether coronary artery bypass grafting or general surgery [36]. In the present trial, it was proven the antiplatelet activity reversal after 12 h and one-day drug cessation was comparable to the generic Ticaloguard® and its brand Brilique®. This will permit a safe cessation of the generic Ticaloguard® when treating it with the same manner and precautions as Brilique®.

Studies suggest a personalized approach that rely on platelet function testing, rather than time since discontinuation, to determine the risk for bleeding complications or to estimate the time needed for platelets to recover is desirable. yet, these approaches require validation in prospective trials [37].The role of preoperative platelet function testing before surgery remains unclear. Hansson et al. highlighted the role of platelet function testing as a valuable tool in timing of surgery in patients with ongoing or recently stopped ticagrelor treatment [37].In the present study interindividual variability in ADP-induced aggregation at different time points after discontinuation of both drugs was noticed, which is in line with the findings of Hansson et al. [38].

The safety profile of the generic Ticaloguard ® was comparable with its brand Brilique®. Several clinical studies have correlated platelet inhibition with bleeding risk [39, 40]. In the present study, no bleeding events were recorded in both generic and brand ticagrelor. Although ticagrelor is generally well tolerated [41], dyspnea which is mild to moderate in severity was observed in premarketing studies [42, 43]. Dyspnea, related to ticagrelor therapy mainly develops shortly after the first days of treatment and has been linked to the increased extracellular level of adenosine [44]. In the present trial, early onset of dyspnea cases was reported only in the brand Brilique®. Minor skin rash cases, resolved spontaneously, were detected in both generic Ticaloguard® and brand Brilique® and were mainly attributed to an allergic reaction to other medication ingredients.

The main limitation of the present study is that it was performed on a group of healthy volunteers who are likely to have a degree of variability in response to ticagrelor compared to patients with ACS. Yet, it may still give grounds for some conclusions regarding generic effectiveness. The study represents an ideal model for proof-of-concept design in building trust in the use of generic drugs in such critical situations.

Future recommendations include testing the generic ticagrelor on a large scale of patients with ACS with a long follow-up period to give a more comprehensive picture of the clinical efficacy and safety of the generic ticagrelor i.e., get real-world data about using generic products in clinical practice.

## Conclusion

To lower drug expenses, maintain drug availability, and ensure quality, it is imperative to promote the use of generic drugs on a large scale. More interventions are needed to improve healthcare members’ knowledge and trust in generic drugs and facilitate ease of entry for the generic drugs into the real market. The clinical efficacy and safety of generic drugs, especially those taken in serious conditions, should be proved. The present study revealed that the antiplatelet inhibition activity and safety of the generic ticagrelor (Ticalogaurd®) are comparable to its brand Brilique® in healthy volunteers. Testing generic drug effects rather than simple bioequivalency, especially for drugs that are used in critical life-threatening situations like ACS is crucial. We advocate applying this form of a clinical trial to test surrogate clinical efficacy for generics that are used in critical indications before having real-world data whenever possible.

## Data Availability

All raw data are available upon request from the corresponding author.

## Abbreviations

ACS: Acute Coronary Syndrome
ADP: Adenosine Diphosphate
CBC: Complete Blood Count
CVDs: Coronary Vascular Diseases
EDTA: Ethylenediaminetetraacetic acid
Hb: Hemoglobin
LD: Loading Dose
LTA: Light Transmission Aggregometer
NSAIDs: Non-Steroidal Anti-Inflammatory Drugs
OTC: Over the Counter Medication
PCI: Percutaneous Coronary Intervention
PLT: Platelet
RCT: Randomized Clinical Trial
PRP: Platelet Rich Plasma
Tica: Ticaloguard
